# Supplementary figures and images for: Global Analysis of Transcription Start Sites in the New Ovine Reference Genome (Oar rambouillet v1.0)
Source: Front Genet. 2020 Oct 23;11:580580. doi: 10.3389/fgene.2020.580580 (PMC7645153; doi:10.3389/fgene.2020.580580)

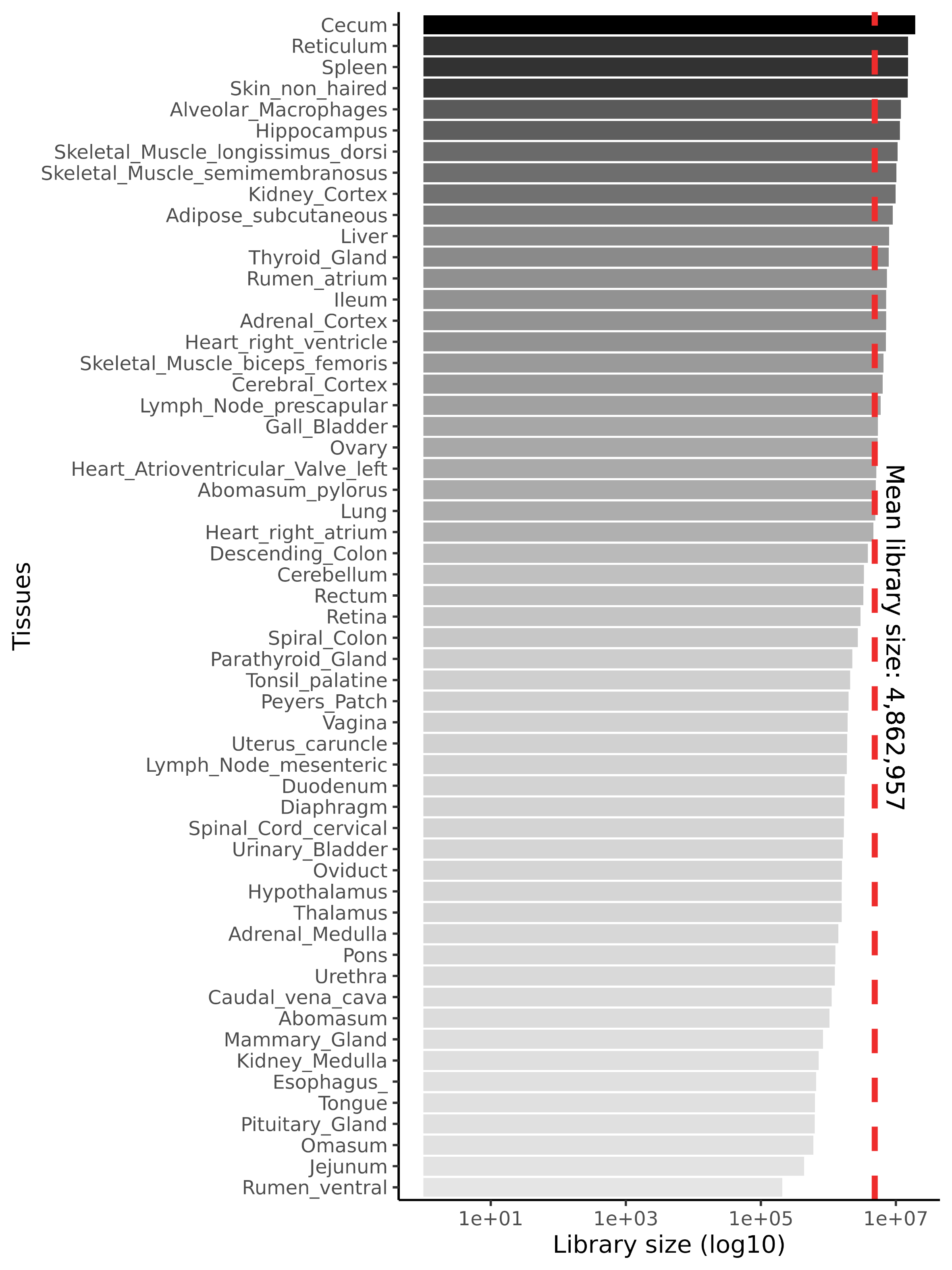

Supplement: Supplementary Figure 1 — CAGE library size for each of the 56 tissues analyzed. [file Image_1.TIF]

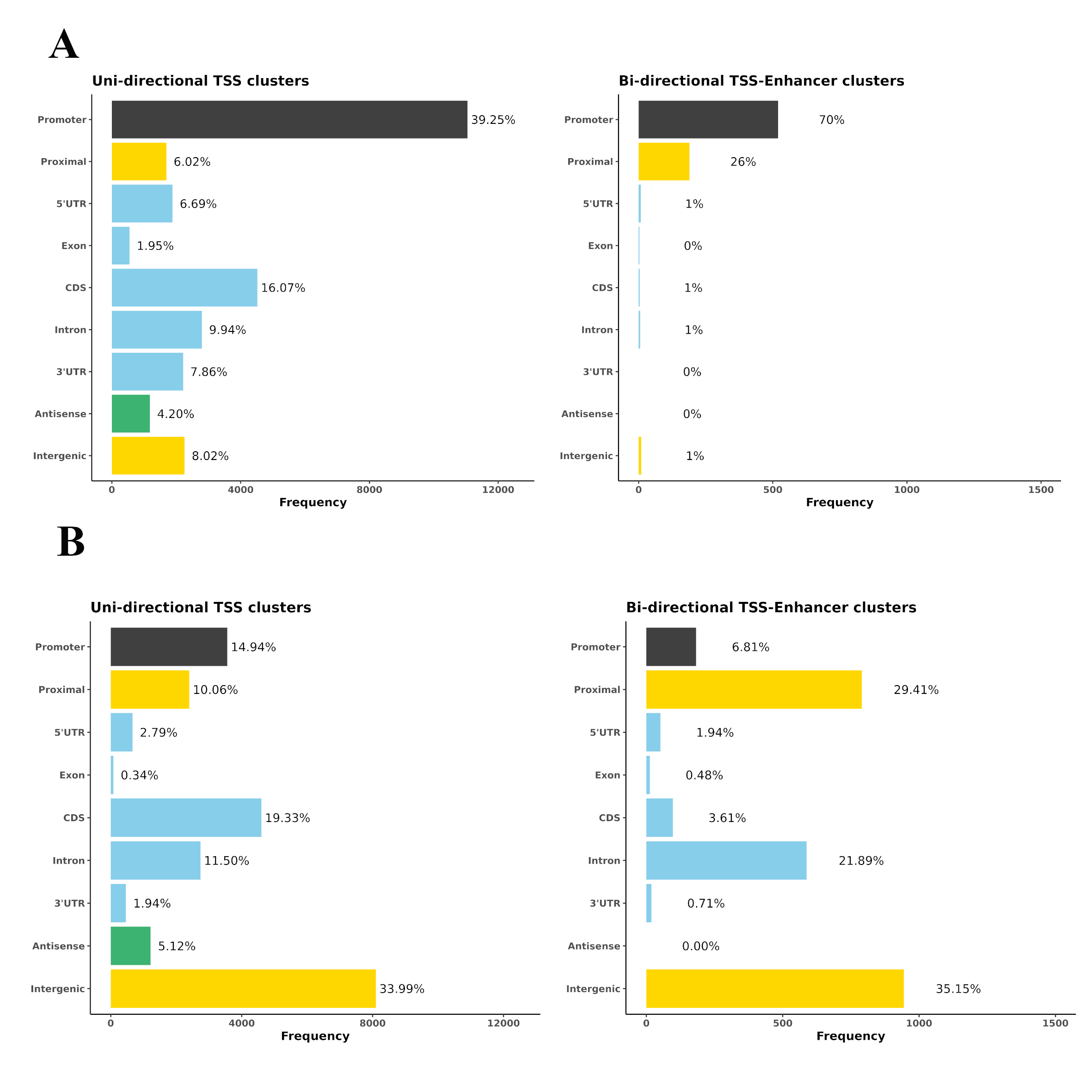

Supplement: Supplementary Figure 2 — The percentage of CAGE tags mapped to each genomic region for Oar rambouillet v1.0 (A) and Oar_v3.1 (B) reference genome assemblies. The counts were averaged across tissues prior to annotation. [file Image_2.TIF]

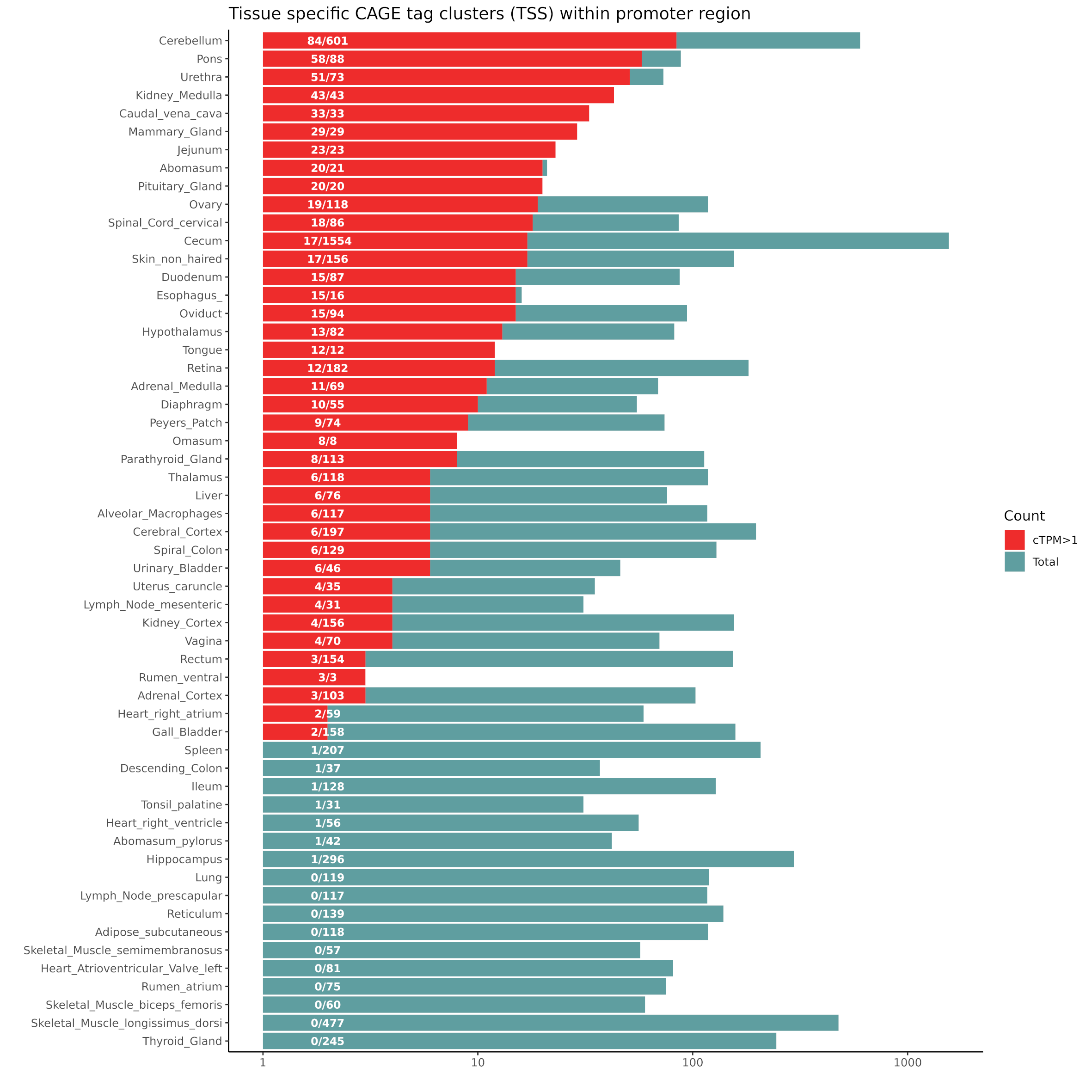

Supplement: Supplementary Figure 3A — The distribution of tissue specific TSS in 56 tissues of Benz2616. The bar shows the count of tissue specific TSS in each tissue with the proportion being expressed with CTPM > 1 colored in red. [file Image_3.TIF]

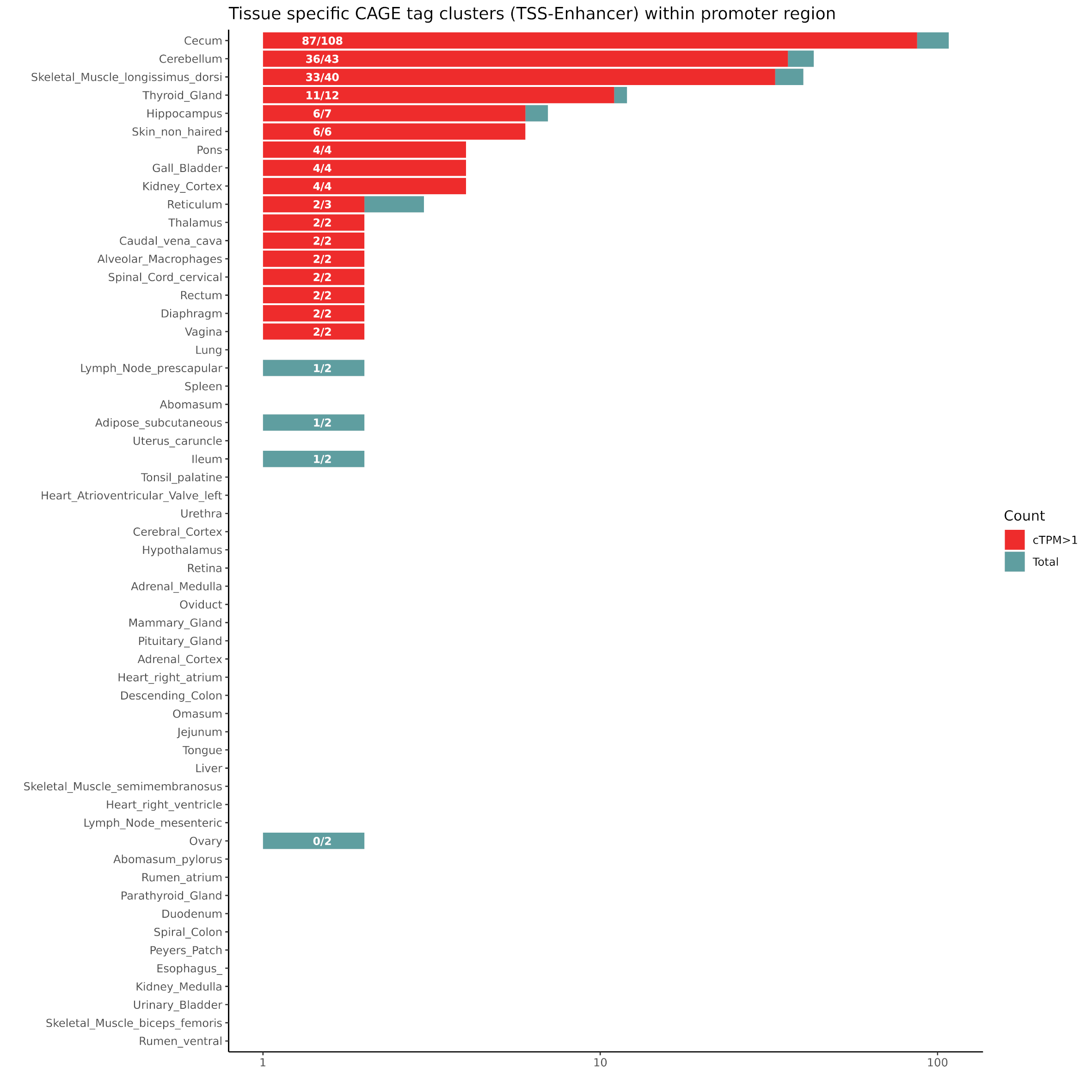

Supplement: Supplementary Figure 3B — The distribution of tissue specific TSS-Enhancers across the 56 tissues from Benz2616. The bars show the count of tissue specific TSS in each tissue with the proportion being expressed with CTPM > 1 colored in red. [file Image_4.TIF]

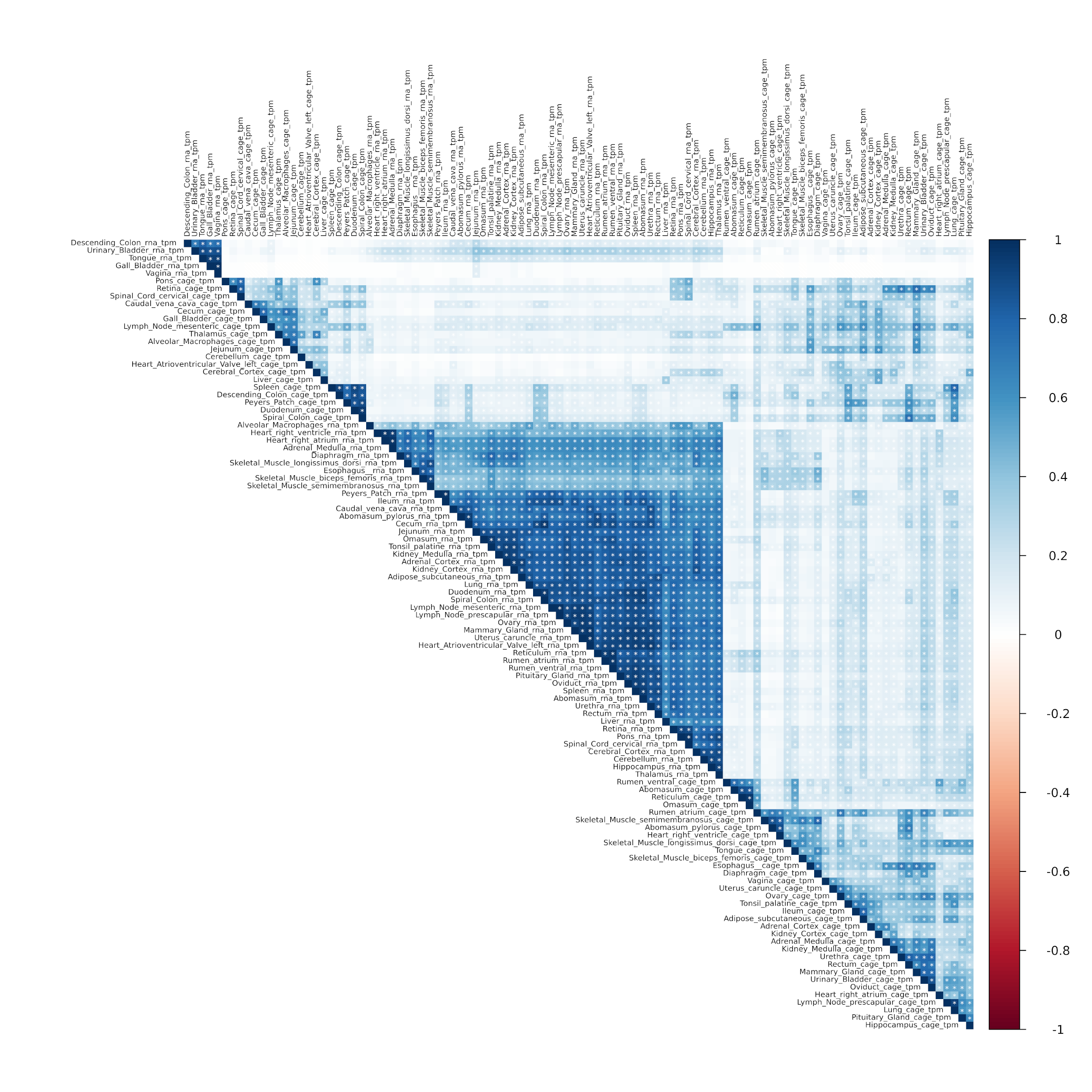

Supplement: Supplementary Figure 4 — Heatmap of mRNA-Seq and CAGE expression profiles (TPM and CTPM). The correlation was calculated over 52 matched tissues and 5732 transcripts—TSS expressed in all tissues. [file Image_5.TIF]

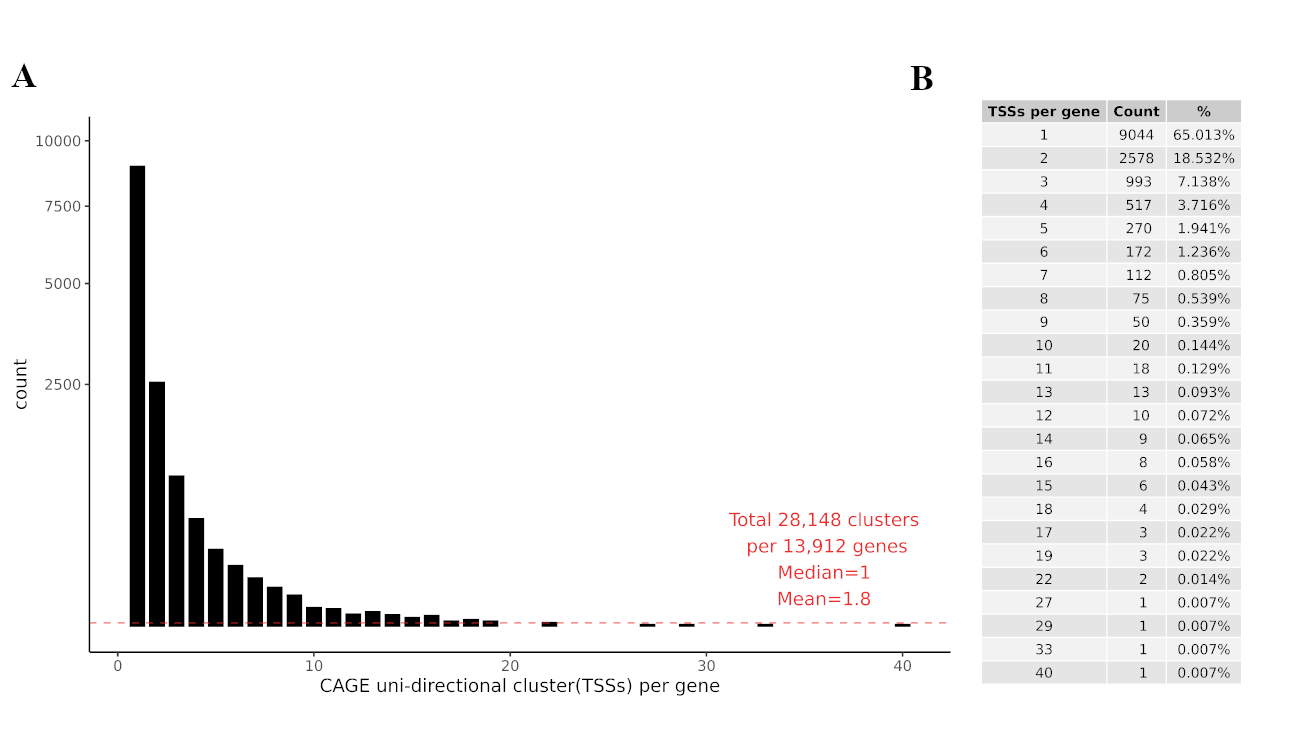

Supplement: Supplementary Figure 5 — Distribution of uni-directional CAGE TSS clusters per annotated gene. (A) The histogram of the TSS cluster per gene. (B) Detailed table of TSS per gene data underlying the histogram and percentage per total TSS clusters. [file Image_6.TIF]
